# Fault Critical Point Prediction Method of Nuclear Gate Valve with Small Samples Based on Characteristic Analysis of Operation

**DOI:** 10.3390/ma15030757

**Published:** 2022-01-19

**Authors:** Zhilong Liu, Jie Liu, Yanping Huang, Tongxi Li, Changhua Nie, Yanjun Xia, Li Zhan, Zhangchun Tang, Lin Zhang

**Affiliations:** 1 School of Mechanical and Electrical Engineering, University of Electronic Science and Technology of China, Chengdu 611731, China; lzluestc@163.com (Z.L.); xiayanjunt@163.com (Y.X.); 2 Institute of Reactor Power Engineering, Nuclear Power Institute of China, Chengdu 610000, China; liujie_cult@163.com (J.L.); hypgroup@163.com (Y.H.); litongxi2021@163.com (T.L.); ch_nie@163.com (C.N.); jellykof@hotmail.com (L.Z.); castalzhang@126.com (L.Z.); 3 Guangdong Institute of Electronic and Information Engineering, University of Electronic Science and Technology of China, Dongguan 523808, China

**Keywords:** fault prediction, Shannon entropy, power spectral entropy, fault critical point, nuclear gate valve

## Abstract

The number of fault samples for the new nuclear valve is commonly rare; thus, the machine learning algorithm is not suitable for the fault prediction of this kind of equipment. In order to overcome this difficulty, this paper proposes a novel method for the fault critical point prediction of the gate valve based on the characteristic analysis of the operation process variables. The operation process of gate valve switch often contains various fault characteristics and information, and this method first adopts the Shannon entropy to describe the power spectrum of vibration signal relevant to the operation process of gate valve switch, and then employs the mean value of the power spectrum entropy as an indirect process variable and further investigates the differences between the indirect process variable under the healthy state and the fault state with a different fault degree. In addition, the power signal of the gate valve is also employed as the direct process variable and the features of the direct process variable under the healthy state and the fault state with different fault degrees are also investigated. Based on the previous indirect process variable and direct process variable, the prediction approach for the critical point of the gate valve fault is established by analyzing the change in the indirect process variable and direct process variable before and after faults. Finally, the data of a nuclear gate valve experiment are employed to demonstrate the feasibility of the proposed method and the results show that the proposed method can effectively predict the fault critical point of the mentioned nuclear gate valve. If the diagnostic threshold is set properly, the critical point prediction of a nuclear gate valve fault can be realized as 100% or close to 100%. Furthermore, the proposed method can be directly applied to the nuclear gate valve in a nuclear power plant to improve the operation reliability of the valve. At the same time, the method can be applied to the fault diagnosis and prediction of valves in other fields, such as the chemical industry.

## 1. Introduction

The requirements for equipment reliability in nuclear power are becoming higher and higher with the development of the nuclear power plants. During the run of the equipment, fault prediction methods can be employed to perform the preventive maintenance of equipment and to further prevent losses resulting from the possible fault of equipment [1,2]. Once a nuclear accident occurs at a nuclear power plant, the consequences will be disastrous and will cause serious damage to the environment and humankind. The nuclear gate valve is the active equipment with the largest proportion of nuclear power plants and its reliability is significant to the nuclear power plants. Therefore, it is of great significance to study the reliability of nuclear valves and fault prediction, which can ensure the safe operation of nuclear power plants and reduce the probability of nuclear accidents.

In recent years, fault prediction methods have been widely continuously investigated. The literature [3] established a diagnosis model based on mathematical calculation methods and predicted fault occurrence by processing and calculating related signals. This method is based on the need for in-depth analysis of each key link of the equipment and understanding its characteristics, then real-time fault prediction is achieved and, finally, achieve real-time failure prediction. However, for large and complex equipment, this method has poor stability. The literature [4] also built a gear system dynamic model and developed a model-based fault feature extraction approach; however, fault prediction was not achieved. The literature [5,6] adopted dynamic Bayesian networks to form a state monitoring and fault prediction method for sensor faults and power transformer faults, effectively solving the problem of sensor and power transformer fault prediction. The literature [7] combined time domain and frequency domain features to propose an adaptive frequency-shift variable-scale stochastic resonance algorithm to predict faults. On the basis of removing signal interference, the time domain and frequency domain characteristics of the signal, such as amplitude, frequency, etc., were extracted, so as to utilize the characteristic value to predict the fault. The advantage of this method is that it can identify the fault location, but the disadvantage is that it is slow. The literature [8] established a BP neural network industrial equipment prediction model based on the dynamic cuckoo algorithm and, finally, realized the effective prediction of industrial equipment fault. The literature [9] proposed a new algorithm which combines the advantages of genetic algorithm and fuzzy logic control algorithm for fault prediction from the perspective of algorithm fusion. The parameters required by the fuzzy logic algorithm are predicted and calculated by the genetic algorithm, and then the fused mathematical model is established and the historical data are used for learning and training. The model obtained after training is the final fault prediction model. The literature [10] took the aerial vehicle as the research object, based on the research and analysis of aerial vehicle data and proposed a data-driven fault prediction method to ensure the reliability. The literature [11] proposed a life prediction model based on Markov theory to identify and predict the state of equipment during operation in real time. Then, the historical data collected by the sensors were analyzed to determine the current state of the equipment and predict the state of the equipment. The model stability is poor during long-term and large-scale application. The literature [12] deeply analyzed the causes of failures and analyzed the relationship between the causes of failures and environmental attributes, then evaluated the impact of failure prediction on overall performance prediction, finally, established a failure prediction model. According to the literature [13,14,15,16,17,18,19], these methods do not need a thorough examination of the mechanism of equipment fault and fall within the black box concept. When the number of samples is insufficient, it is difficult to establish an accurate fault prediction model. In the literature [13,14,15] combined system identification theory and system optimization theory and established fault prediction models based on Bayesian, ion filtering, time series and deep learning. In the literature [16,17,18], neural network technique for machinery fault detection and life prediction is shown, which improves accuracy. In the literature [19], a neural network bearing life prediction model based on health indicators is proposed for sensitive feature quantity. The time-frequency feature set is constructed by using monotonicity and correlation, which solved the problem that the fault threshold is difficult to determine. The literature [20] proposed a fault prediction expert system, which is based on algorithms of subtractive clustering and human-computer interaction. Through logical reasoning and symbolic information, as well as the knowledge and experience of dealing with equipment fault, the status of equipment is identified, diagnosed and predicted. This prediction system solves the bottleneck problem of knowledge acquisition in the expert system and optimizes the construction speed of the expert system. The literature [21] proposed a fault prediction method based on logic decision tree by comparing various algorithms in machine learning and statistics, which effectively solved the problem of model selection for different fault data sets based on model-based fault prediction technology. In the literature [22], a fault prediction and diagnosis method based on quantitative wavelet and improved LDB algorithm is proposed. Firstly, a quantitative wavelet basis for fault analysis is proposed by using information entropy and then, for the problems of LDB itself, an optimal wavelet basis decomposition algorithm based on the improved local discriminant basis theory is proposed, which finally improves the fault recognition rate and reduces the computational complexity. The literature [23] proposed a fault prediction method based on fuzzy neural network based on peak density. After experimental analysis, this method has faster convergence speed and higher accuracy than existing methods, but it is difficult to build a predictive model for a complex and multiple system. The literature [24] proposed a combination of fault prediction methods based on gray prediction and Kalman filtering, established a mathematical model, analyzed and processed historical data, predicted and identified the operating parameters of the equipment and then judged the failure trend. In order to analyze the relationship between different variable parameters, the algorithm needs to process a large amount of historical data. In the literature [25], the weather is regarded as the variable of fault prediction model. By constructing Bayesian network based on causality and inputting characteristic data for dimension reduction, the fault prediction of railway related system is achieved and the fault of railway related system is avoided. The literature [26] proposed a data-driven failure prediction method based on probability density estimation. The method introduced an abnormality index to measure the abnormality of the sample and then improved the constraint conditions to effectively reduce the scale of the objective function and the number of solutions. This method greatly improves the computational efficiency of the model. The literature [27] presented an approach to early fault prediction of circuits. The proposed method uses fast Fourier transform (FFT) to obtain the fault frequency signature, principal component analysis (PCA) to obtain the most important data with reduced dimension and a convolutional neural network (CNN) to learn and classify the fault. It provides a fault prediction accuracy of 98.93% and 98.91% for comparator and amplifier circuits, respectively. In the literature [28], based on the K-means clustering analysis method of fault case big data machine learning, the fault identification of rotating machinery without the support of external experts is studied, which improves the accuracy of fault diagnosis. In the literature [29], a fault prediction method based on knowledge prediction time series is proposed. Two neural network models are used to realize multi-dimensional feature sequence prediction and waveform prediction; finally, fault prediction is realized by associating fault diagnosis knowledge. In the literature [30], an early defect prediction model based on wavelet packet decomposition and dynamic kernel principal component analysis (wpd-dkpca) is investigated using machine learning technology to fulfill the demands of engineering applications. In the literature [31], to maintain the objectivity of prediction model, the multi-objective particle swarm optimization technique and random walk strategy are employed to optimize long-term and short-term memory (LSTM) network. The literature [32] provided a detection index based on residual SMD (square of the Mahalanobis distance) that could diagnose but not predict faults. The literature [33] used neural network to realize fault-tolerant control of fractional time-delayed Systems and achieved good results. This method can improve the system reliability. This literature involves theoretical details, which can be referred to in [34]. It can be seen from the above literature that most of the current failure prediction research is based on two directions, the first is a fault prediction approach based on data and machine learning, the second is a model-based fault prediction method. Failure prediction model based on data and machine learning does not require a good understanding of the failure mechanism of the system as long as the data are released, but it is affected by the error of the collected data and the number of data samples. Model-based fault prediction methods are characterized by high accuracy, but it is difficult to model complex fault systems.

From the standpoint of the nuclear valve’s sensitive parameter characteristics, operating characteristics and fault characteristics, this paper proposes a gate valve fault critical point prediction method based on the analysis of the characteristics of operating process variables, in view of the extremely small fault samples of the new nuclear valve and the machine learning algorithm is not suitable for such equipment. The writing method of some contents of the article refers to the literature [35]. Our main contributions of this paper are demonstrated as follows:

(1) Taking each opening and closing process of the gate valve as the research unit, use Shannon entropy to measure the power spectrum of the vibration signal during the opening and closing process of the gate valve. The mean value of power spectrum entropy is calculated and utilized used as an indirect process variable, then analyze the change characteristics of the mean value of power spectrum entropy when the gate valve is healthy and when the gate valve has different degrees of failure.
(2) Considering the gate valve power signal as the direct process variable to analyze the characteristics of the power signal change before the gate valve failure and under different failure degrees.
(3) The critical point of gate valve fault is predicted by analyzing the characteristic changes of direct process variable and indirect process variable.
(4) Finally, based on nuclear gate valve experimental analysis, the method is verified by experiments. The experimental results show that the method can effectively predict the critical point of nuclear gate valve failure.

The gate valve fault prediction method proposed in this paper has good stability and can avoid the occurrence of fault misdiagnosis, because the fault prediction result is jointly determined by frequency domain characteristics and time domain characteristics. Meanwhile, it overcomes the problem of extremely small failure samples of the new nuclear gate valve.

The rest of this paper is organized as follows. Section 2 investigates the characteristics of the vibration signals and introduces the proposed prediction method of fault critical point for gate valve based on the operation process variables. Section 3 employs vibration signals of practical nuclear gate valve to demonstrate the proposed method. Section 4 compares the proposed method with the existing typical methods. Conclusions are given in Section 5.

## 2. Critical Point Prediction Method for Gate Valve Failure

Here, we will propose an efficient prediction method for the fault critical point of gate valve. First, we will analyze the vibration signals and power signals of gate valve under different states, i.e., healthy state and fault states with different fault degrees and we will also propose two kinds of state variables including direct variable and indirect variable to identify the current state of the gate valve. Then, an efficient integrated prediction technology is proposed to predict the state of the gate valve.

### 2.1. Vibration Signals of Gate Valve and In-Direct Variable

**Figure 1:**
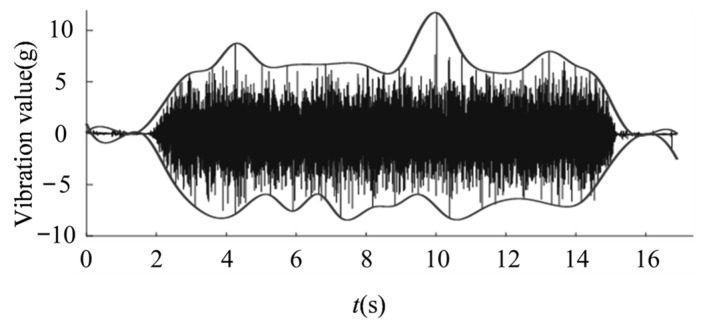
Vibration signal diagram when the gate valve is normally closed.

**Figure 2:**
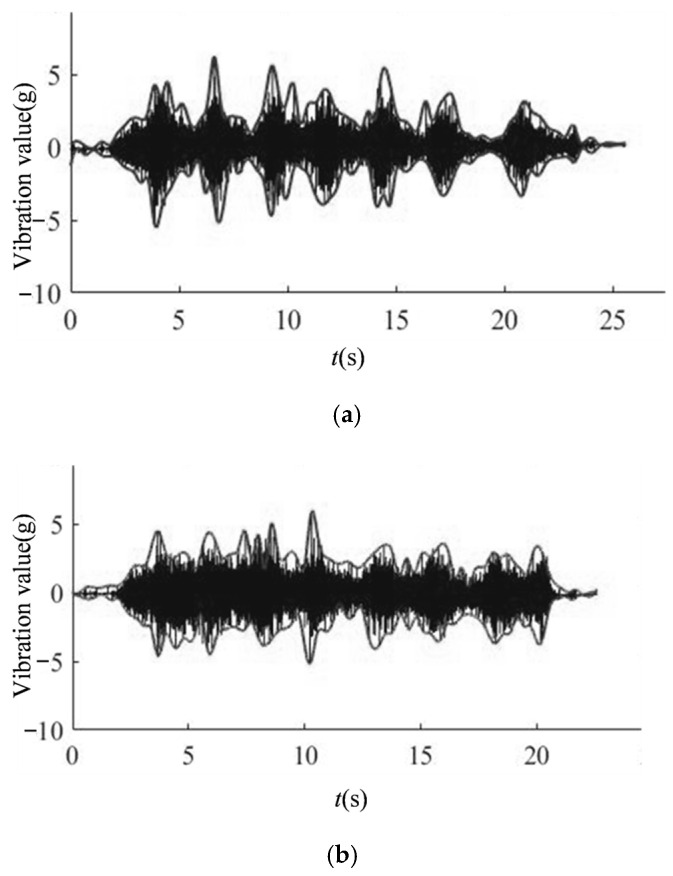

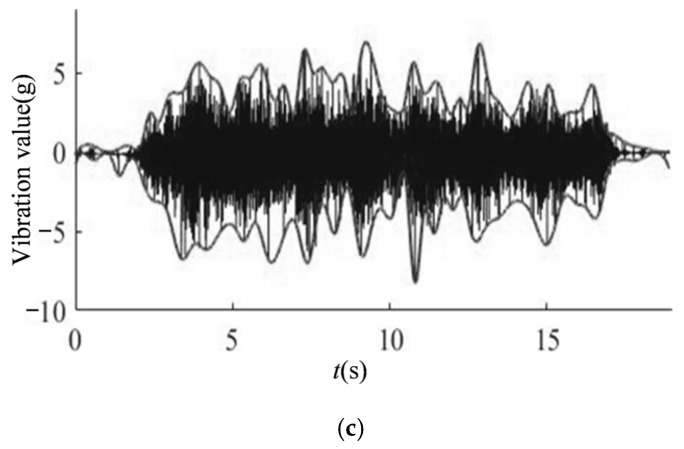
Vibration signal diagram of the three valve closing processes before gate valve failure. (**a**) The first closing process of gate valve before failure. (**b**) The second closing process of gate valve before failure. (**c**) The third closing process of gate valve before failure.

Different degrees of gate valve failure, vibration signals exhibit different variation characteristics. Figure 1 is the vibration signal diagram when the gate valve is closed normally and Figure 2 is the vibration signal diagram of three times closing process before gate valve failure. In order to observe and analyze the change characteristics of the vibration signal more clearly, the waveform envelopes of the vibration waveforms in Figure 1 and Figure 2 are drawn. By comparing normal vibration waveform and three vibration waveforms before the failure, it can be seen intuitively that the gate valve vibration signal has obviously changed regularly. As gate valve gets closer to failure, vibration signal changes more obviously. The power spectrum entropy of the vibration signal in the valve switching process represents the distribution of vibration energy at each frequency. If energy distribution is more even in each frequency, signal uncertainty is greater and power spectrum entropy is greater. If energy distribution is more uneven in each frequency, signal uncertainty is smaller and power spectrum entropy is smaller. Based on this, in order to more intuitively characterize the change law of vibration signal, power spectrum entropy is used as an indirect process variable to describe the change characteristics of vibration signal. As vibration signal law becomes more and more obvious in the several operation processes before valve failure, the entropy value of the vibration signal power spectrum of the gate valve operation processes before failure will continue to decrease.

Shannon entropy characterizes the quantitative measurement of information and it is also an evaluation index of the uncertainty of information. The random process or random variable characterizes the uncertainty of information. *Y* is a random variable assumed and its probability distribution is *P*{*Y = y_i_*} = *p_j_*, where *j* ranges from 1 to *n*, then calculation formula for entropy *S* is:

(1) $$S(Y)=-\sum_{j=1}^{n}p_{j}\log_{a}(p_{j})$$

where *a* is the logarithmic base.

Assuming that the number of sensors installed in the valve body is *z*, the vibration signals of gate valve during each valve opening or closing process is *X_i_*(*t*), 0 < *i* ≤ *z*. The frequency domain signal *X_i_*(*w*) is obtained by Fourier transform, then power spectrum is:

(2) $$S_{i}(w)=\frac{1}{2\pi n}{\left|X_{i}(w)\right|}^{2}$$

The power spectrum *S_i_*(*w*) can be regarded as composed of *S*_*i*1_(*w*), *S*_*i*2_(*w*), …, *S_in_*(*w*), then the power spectrum entropy *M* is calculated by the formula:

(3) $$M_{i}=-\sum_{j=1}^{n}C_{ij}\log_{a}(C_{ij})$$

where *C_ij_* is the ratio of the *j*-th power spectrum component to the total power spectrum for a certain vibration sensor.

Each time gate valve is opened and closed, power spectrum entropy *M_in_* can be calculated based on the data collected by single-channel vibration sensor, *n* is the *n*-th valve opening or closing and *i* is the *i*-th sensor. Then, power spectral entropy matrix *M_in_* is obtained. In the power spectrum entropy matrix *M_in_*, data in each column represent power spectrum entropy calculated when valve is opened or closed at a certain time. Each column of different data represents power spectrum entropy calculated based on different acceleration sensors. Each row of power spectral entropy matrix *M_in_* represents power spectral entropy calculated based on the same sensor. Different elements in each row represent the power spectral entropy of different valve opening or valve closing.

(4) $$M_{in}=\left[\begin{array}{cccc} M_{11} & M_{12} & \ldots & M_{1n}\\ M_{21} & M_{22} & \ldots & M_{2n}\\ \ldots & \ldots & \ldots & \ldots \\ M_{z1} & M_{z2} & \ldots & M_{zn} \end{array}\right]$$

In order to analyze the overall characteristic changes of gate valve vibration signal, each valve operation process is regarded as an object, then average power spectrum entropy *M_a_* is calculated, that is, the average value of each column of matrix *M_in_* is calculated.

(5) $$M_{a}=\frac{\sum_{i=1}^{z}M_{in}}{z}$$

When the severity of the fault continues to increase, the vibration signals law of valve switching process becomes increasingly obvious, vibration energy is increasingly unevenly distributed at each frequency and power spectrum entropy value should have a decreasing trend. When power spectrum entropy value is small to a certain extent, it is considered that there may be a potential failure probability. Then, fault diagnosis variable *L* is defined.

(6) $$L(n)=\{\begin{array}{cc} 1 & M_{a}(n)\leq k_{1}\\ 0 & others \end{array}$$

where *n* represents the *n*-th time valve is opened and closed and *k*_1_ is threshold, which is a value greater than zero.

When the value of the diagnostic variable *L*(*n*) is 1, it is considered that there may be a potential failure trend. The application environment of valve is different, noise is different and specific noise environment has specific impact on the distribution of vibration energy. Therefore, the selection of threshold *k*_1_ is based on experimental experience. Valve opening and closing action experiment is repeated in the application environment to determine the impact of environmental noise on vibration energy distribution and combined with vibration energy distribution in the case of failure, the selection of the threshold *k*_1_ is determined.

The above diagnostic method based on the indirect variable is judged as one that can only obtain potential fault trend and cannot accurately predict critical point valve failure. Therefore, an auxiliary diagnostic variable is needed to assist diagnosis method based on power spectrum entropy to accurately predict the critical point of valve fault. In Section 2.2, the valve power signal is proposed as a direct variable to assist the valve failure critical point.

### 2.2. Operation Power Signal of Gate Valve and Direct Variable

**Figure 3:**
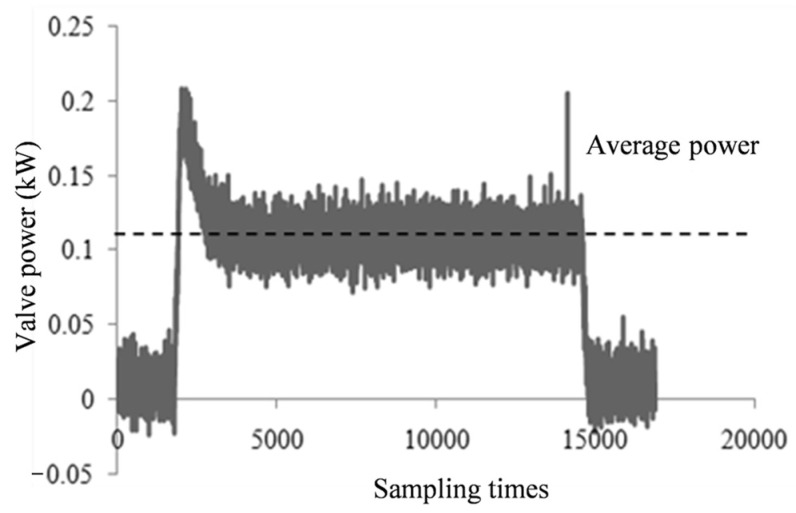
Power waveform when the gate valve is normally closed.

**Figure 4:**
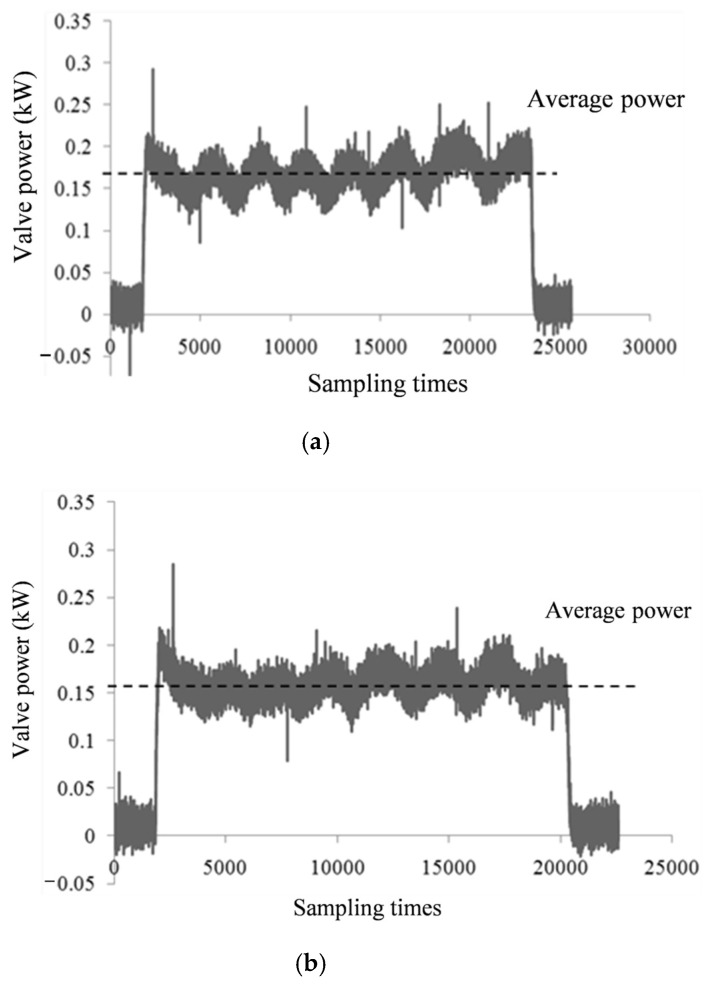

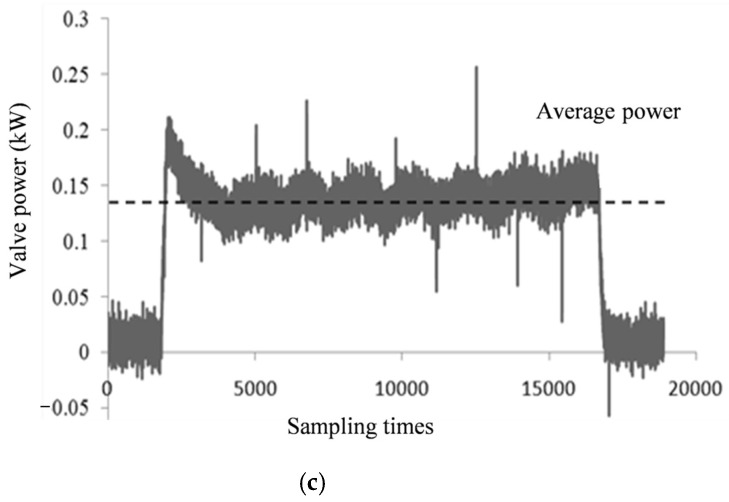
The power waveforms of three valve closing processes before gate valve failure. (**a**) The first closing process of gate valve before failure. (**b**) The second closing process of gate valve before failure. (**c**) The third closing process of gate valve before failure.

Different degrees of gate valve failure, power signal exhibits different variation characteristics. Figure 3 shows the power waveform of the gate valve in normal operation under fixed operating condition, Figure 4 shows the power waveforms of three valve closing processes before gate valve failure under the same operating condition. Through the comparison of waveforms, it can be seen that power increases in the case of gate valve failure, and as the severity of fault is different, the degree of power increase is also different.

When gate valve is in normal operation, operating power is not only related to the rated power, but also related to working conditions. The operating power of gate valve is a variable and the size of the variable value is not fixed. In addition, the operating power varies within a certain range with different operating conditions. In the change process of normal temperature conditions to high temperature and high pressure conditions, gate valve power gradually rises, so the power of gate valve during operation process is not suitable for the direct application of gate valve fault diagnosis and fault prediction. In this paper, the operating power of gate valve is used as a direct process variable to assist the prediction of fault critical point.

Define the failure prediction auxiliary variable *d*(*n*):

(7) $$d(n)=\frac{\sum_{i=1}^{m}P_{i}}{m}$$

Among them, *n* is the *n*-th time to open or close valve, *i* is the *i*-th operation data collected and *d*(0) is the variable under normal conditions of gate valve.

As shown in Formula (6), when gate valve is opened and closed for the *n*-th time, if obtained power rise judgment variable *R* is greater than threshold *k*_2_, it is considered that the power of gate valve is increasing during the *n*-th operation. At this time, the value of the power rise judgment variable *R* is 1, otherwise it is 0.

(8) $$R(n)=\{\begin{array}{cc} 1 & \frac{d(n)\;-\;d(0)}{d(0)}\geq k_{2}\\ 0 & others \end{array}$$

After the *n*-th gate valve action, if power does not change, (*d*(*n*) − *d*(0))/*d*(0) is close to 0. Therefore, the minimum value of the rate of change (*d*(*n*) − *d*(0))/*d*(0) is close to 0; If power changes, then the maximum value of (*d*(*n*) − *d*(0))/*d*(0) is (*P_s_* − *P_h_*)/*P_h_*, among them, *P_s_* is the power of gate valve locked-rotor and *P_h_* is the power of gate valve under normal operation. Therefore, the value range of *k*_2_ is 0 < *k*_2_ < (*P_s_* − *P_h_*)/*P_h_*, at the same time, the selection of threshold *k*_2_ should fully consider grid voltage fluctuations and valve load disturbances and the value is generally greater than 0.1.

The above analysis gives the characteristics of power signal variation under different fault degrees and explains why the power signal cannot be used for fault diagnosis and fault prediction directly. Therefore, the power signal is used as a direct diagnostic variable to assist fault critical point prediction. In Section 2.3, the prediction method of fault critical point—based on the combination of direct variable and indirect variable—is given.

### 2.3. Fault Critical Point Prediction Method Based on Direct Variable and Indirect Variable

In Section 2.1 it is shown that the method based on indirect variables cannot accurately predict the critical point of gate valve fault. The direct variables proposed in Section 2.2 are not suitable for direct fault diagnosis or fault prediction, but can be used as auxiliary fault prediction variables. Therefore, a prediction method of valve fault critical point based on the combination of direct variables and indirect variables is proposed.

**Figure 5:**
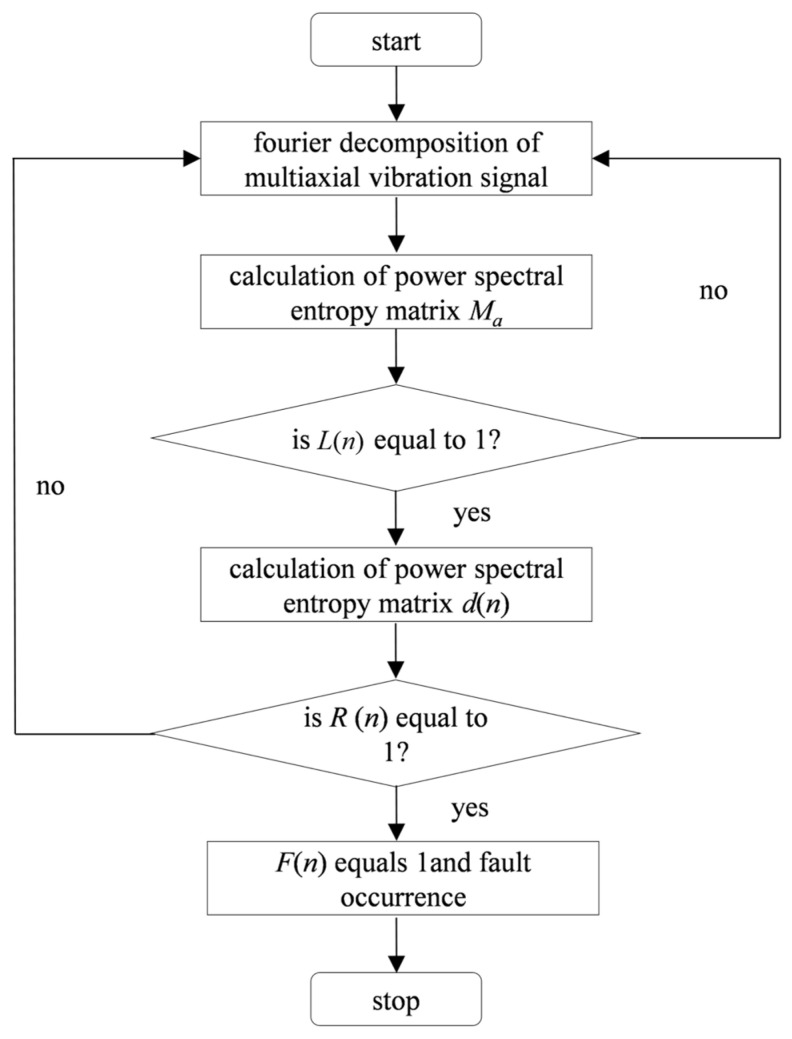
Overall block diagram of gate valve fault critical point prediction method based on analysis of characteristics of operating process variables.

Figure 5 is the overall block diagram of the gate valve fault critical point prediction method based on the analysis of the characteristics of the operating process variables. Taking each opening process or closing process of gate valve as the research unit, the vibration signals of gate valve switching operation is measured by the vibration sensor from gate valve body, then the vibration signals is Fourier transformed, average power spectrum entropy is calculated as an indirect process variable and the power spectrum entropy trend is analyzed to obtain the fault area and the non-fault area. The power signal of gate valve measured by power sensor is regarded as a direct process variable and the real-time change of power signal is analyzed. Combining the analysis of direct process variables and indirect variable characteristic changes, the fault criticality is predicted. In the next section, through the gate valve fault experiment, the proposed method is demonstrated.

When the average power spectrum entropy of gate valve decreases to a certain value, variable *L*(*n*) is 1 and when the power of gate valve increases to a certain value, variable *R*(*n*) is 1. At this time, it is considered that the *n*-th valve action is a critical fault and the predictive variable *F* of critical fault point is 1 and *F* is defined as follows:

(9) $$F=L\&R=\{\begin{array}{cc} 1 & critical\;fault\;point\\ 0 & others \end{array}$$

## 3. Fault Prediction Experiment and Result Analysis

**Figure 6:**
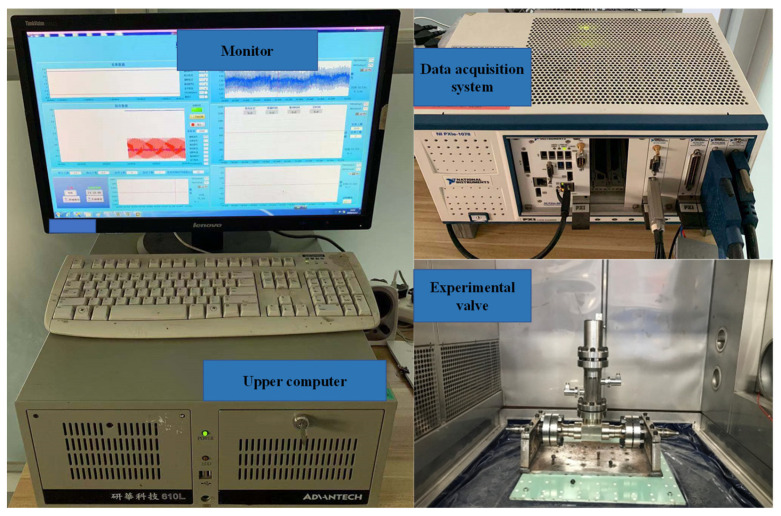
Experimental platform for nuclear gate valve.

The electric actuator of the nuclear grade gate valve is a three-phase asynchronous motor. The line voltage for opening the valve is 380 V AC, and the line voltage for closing the valve is 220 V AC; the rated current of the switch is 2.19 A/1.26 A, respectively. Figure 6 is the experimental platform of nuclear gate valve. It can be seen that it mainly includes upper computer, industrial control computer, data acquisition system and experimental valve. The acceleration sensor used in the experiment is a high temperature resistant vibration sensor, the model is 2273am1; the experimental data acquisition system is the PXI data acquisition system; the data acquisition is carried out at a frequency of 1000 Hz during the experiment and enters the data acquisition analog quantity acquisition board through the charge amplifier. High temperature and high pressure were selected as the experimental conditions for the on–off valve fault experiment of gate valve, and the specific conditions were 260 °C and 13.7 Mp.

**Figure 7:**
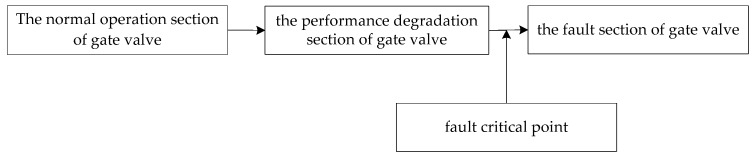
Connection of three operation sections and location of fault critical point of nuclear gate valve.

The working part of a nuclear gate valve is separated into three sections: the normal operation section of gate valve, the performance degradation section of gate valve and the fault section of gate valve. The gate valve runs normally without performance deterioration in the normal operation portion; the gate valve has a little defect in the performance degradation range, but it can still operate; and the gate valve may fail at any moment in failure region. The adjacent points between the fault area and the performance degradation area of the nuclear gate valve are the fault critical point. Figure 7 depicts the progression of three regions, as well as the position of fault critical location.

The vibration signal characteristics of several previous actions (closing process or opening process) of the gate valve fault are very obvious and the waveform is very close. The fault may occur at any time during these actions. Therefore, the previous actions of the valve fault are identified as the fault area.

**Figure 8:**
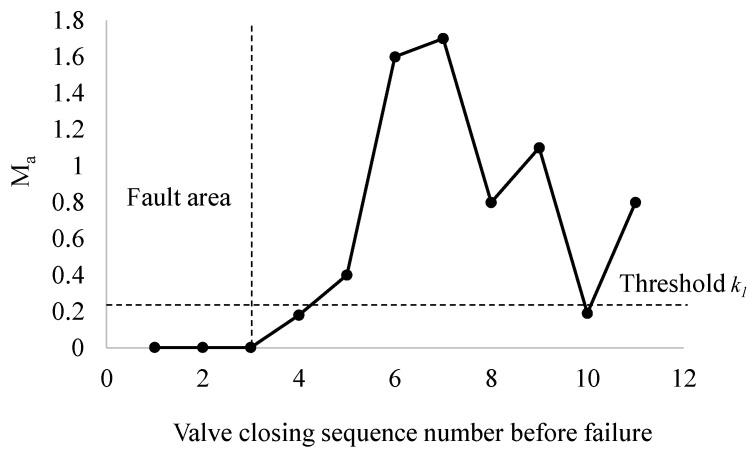
Variation trend of gate valve power in eleven valve closing processes before fault.

Figure 8 shows a variation trend of the mean value *M_a_* of power spectral entropy in the 11 valve closing processes before the fault. It can be seen that the mean value *M_a_* of the power spectral entropy in the three valve operation processes before the fault is very close and that the value is close to 0. At this time, the vibration signal shows obvious regular characteristics. Therefore, the valve operation from the first time to the third time before the fault is the fault area; the fourth time before the fault is the fault critical area, and the rest are non-fault areas. In the non-fault area, the power spectrum entropy of the gate valve fluctuates due to the environmental vibration and other factors.

**Figure 9:**
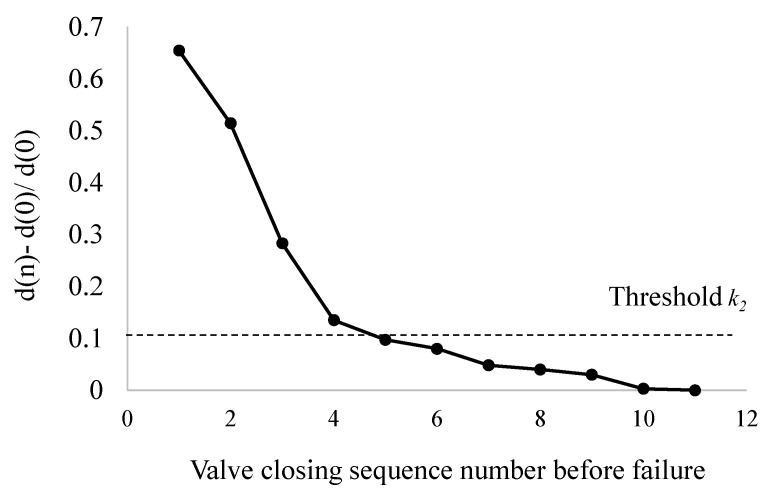
The change trend of (*d*(*n*) − *d*(0))/*d*(0) in eleven valve closing processes before fault.

Figure 9 shows the change trend of (*d*(*n*) − *d*(0))/*d*(0) in the eleven valve closing processes before the fault. It can be seen that (*d*(*n*) − *d*(0))/*d*(0) in the four valve operation processes before the fault increases significantly, indicating that the power in the fault area increases significantly. Before the failure, the fourth valve closing process (*d*(*n*) − *d*(0))/*d*(0) is greater than the threshold *k*_2_, which indicates that there may be a failure trend in this operation.

**Figure 10:**
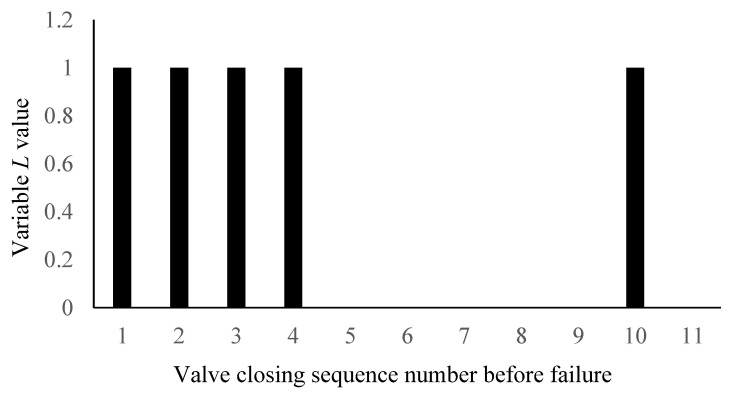
The change diagram of fault diagnosis variable *L*.

Figure 10 shows the change diagram of the fault diagnosis variable *L*. It can be seen that the *L* value changes from 0 to 1 in the 1th, 2th,3th, 4th and 10th valve closings and the variable *L* value is 0 in the other valve closing. This is due to the fact that the mean value of power spectral entropy is less than the threshold *k*_1_ at the 1th, 2th,3th, 4th and 10th valve closings, and the mean value of power spectral entropy is greater than threshold *k*_1_ at the other valve closings.

**Figure 11:**
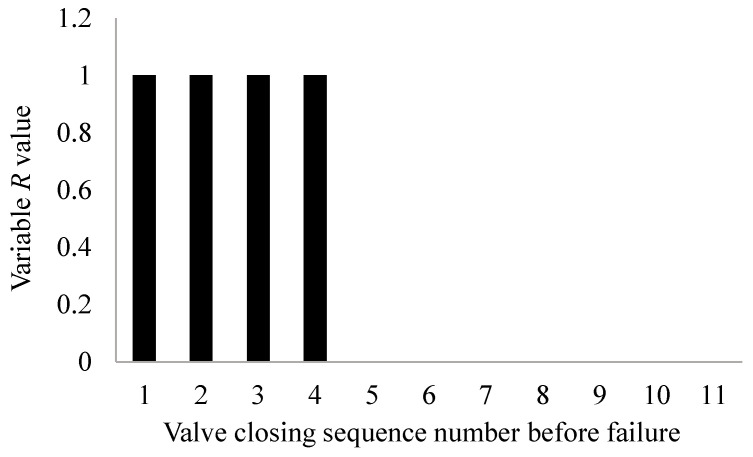
The change diagram of fault diagnosis variable *R*.

Figure 11 shows the change diagram of fault diagnosis variable *R*. It can be seen that when the valve is closed for the first time to the fourth time before the gate valve fault, compared with the power value during normal operation of the valve, because the power value increases greatly, (*d*(*n*) − *d*(0))/*d*(0) is greater than the threshold *k*_2_, so the variable *R* value changes from 0 to 1 and the variable *R* value is 0 when the valve is closed for the other times.

**Figure 12:**
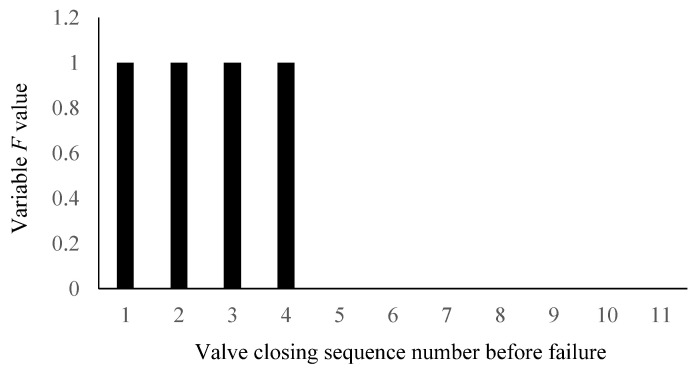
The change diagram of predictive variable *F* at critical point of failure.

Figure 12 shows the change diagram of the predictive variable *F* at a critical point of failure. It can be seen that when the valve is closed for the fourth time before the failure, the value of the variable *F* changes from 0 to 1 and it is successfully predicted that the valve is closed for the fourth time before the failure as the critical point of the gate valve failure.

## 4. Comparative Analysis with Typical Methods

The structure of the nuclear gate valve is relatively complex and it is difficult to establish a parameter model for fault prediction. Therefore, the model-based fault prediction method is generally not selected to predict the fault of a nuclear gate valve or mechanical equipment. The fault prediction method based on data-driven or machine learning can implement fault prediction for equipment with high accuracy in the case of a large amount of feature data, such as in the literature [13,14,15,16,17,18,19]; such methods do not pay attention to the fault mechanism and fault characteristics of the equipment, or simply pay attention to the fault signal characteristics, focusing on the number of fault characteristic data and samples. A large number of fault characteristic data and rich fault samples can establish accurate data fault model or machine learning fault model prediction, so as to achieve high fault prediction accuracy. The methods are not only affected by the number of fault characteristic data and samples, but also can-not obtain an accurate fault prediction model if the data are affected by accidental environmental noise.

The fault critical point prediction method of nuclear gate valve based on multi sensitive operating parameter feature fusion proposed in this paper is based on the analysis of its own characteristics, operating characteristics and mechanism. The new nuclear grade gate valve has a small sample and has the characteristic of intermittent operation. It only needs to be operated when the valve is open or closed. It only needs to run when the valve needs to be opened or closed. During normal operation, the change characteristics of *x*-axis vibration signal, *y*-axis vibration signal, *z*-axis vibration signal and electrical signal have certain rules. In the case of a fault, the overall characteristics of these variables will change regularly. These changes are caused by the clamping and impact of internal mechanical components of the nuclear gate valve. The method proposed in this paper does not need a large number of fault samples or a large number of fault characteristic data, but it needs to carefully select the diagnosis threshold. The discussion of the diagnosis threshold has been given above. Before the application of this method, it needs to be debugged many times in the use environment according to the threshold selection principle in the use site, so as to select the best threshold. Table 1 shows the comparative analysis of typical equipment fault prediction methods and the methods in this paper. By comparison, the method proposed in this paper can achieve higher accuracy, does not rely on a large number of data and samples and can achieve fault prediction.

## 5. Conclusions

In this paper, a fault critical point prediction method of a nuclear gate valve based on the multi sensitive parameter fusion of operation process is proposed. Combined with the operation characteristics of the nuclear gate valve, the variation characteristics of sensitive parameters before and after faults are analyzed, and the specific details of fault critical point prediction method are described. The proposed method is verified in the fault experiment of the nuclear gate valve. Multiple samples of the gate valve are tested and the experimental results of multiple samples are consistent, which effectively predicts the fault critical point of the nuclear gate valve.

(1) For the new nuclear gate valve, the proposed fault critical point prediction method can identify almost 100% of the fault critical point if the diagnostic threshold is selected appropriately. Compared with traditional methods based on variable analysis or machine learning, it improves the accuracy of fault identification to a certain extent.
(2) The accuracy of the proposed fault critical point prediction method depends on the selection of diagnosis thresholds. The selection principle of diagnosis thresholds is given in this paper. In the worst case, if the threshold selection is inaccurate and the critical point prediction is inaccurate, the fault can still be diagnosed in advance to a certain extent.
(3) In the case of small samples, this method can effectively predict the fault critical point of the new nuclear gate valve, which is of positive significance to the operation and maintenance of the gate valve and improves the reliability of the nuclear power plant. At the same time, this method can also be applied to the fault critical point prediction of valves in other fields, such as the chemical industry.

## Figures and Tables

**Table 1:** Comparison of typical equipment fault prediction methods and method in this paper.

Existing Typical Fault Prediction Method	Method Accuracy	Method Advantages	Method Disadvantages	Remarks
Method based on canonical variate analysis (CVA)	≤91.4%	A large number of samples and fault characteristic data are not required for model training	Fault diagnosis is realized, fault prediction is not realized	Based on the analysis of typical variables, details are given in literature [31]
Method based on clustering and data	80–94%	High diagnostic accuracy	A large number of data and samples are required and failure can-not be predicted	It belongs to the method of machine learning, details are given in literature [28]
Sensitive variable feature fusion analysis method proposed in this paper	If the threshold is selected appropriately, accuracy is 100%	Small amount of calculation, high accuracy, no need for a lot of data and samples	Need to choose thresholds	According to the given principles, appropriate thresholds can be selected. If thresholds is not accurate, fault can still be predicted to a certain extent

## Data Availability

Not applicable.
